# Creating a novel algorithm for studying the strong convergence to a sequence with applications

**DOI:** 10.1371/journal.pone.0319047

**Published:** 2025-04-15

**Authors:** Hasanen A. Hammad, Mohammed E. Dafaalla, Manal Elzain Mohamed Abdalla

**Affiliations:** 1 Department of Mathematics, College of Sciences, Qassim University, Buraydah, Saudi Arabia; 2 Department of Mathematics, College of Science and Arts, King Khalid University, Mahayil, Saudi Arabia; King Abdulaziz University Faculty of Sciences, SAUDI ARABIA

## Abstract

This manuscript introduces a novel algorithm tailored to investigate the strong convergence of sequences under specific conditions. Notably, the framework incorporates a finite family of generalized demimetric operators within real Hilbert spaces, broadening existing operator theory. The proposed algorithm efficiently establishes strong convergence and demonstrates its versatility through successful applications, including proving the existence of solutions to split minimization and feasibility problems, thereby showcasing its potential in optimization and numerical analysis.

## 1 Introduction

Determining zero points of maximal monotone operators (MMOs) is a fundamental aspect of optimization, underpinning solutions to various practical problems across disciplines and driving theoretical advancements. Notably, this concept facilitates efficient reformulations of prominent mathematical challenges, including split feasibility, variational inequalities, and convex minimization problems. See [1–8] for more details.

This is one way to formulate the problem mathematically:


$$\begin{array}{ccc} \text{find }\mu \in ℜ\text{ such that }0\in B\mu & & \end{array}$$ (1)

where $B:ℜ\to 2^{ℜ}$ is a MMO described on a Hilbert space (HS) *ℜ* .  The symbol $B^{-1}(0)$ represents the set of solutions of the problem (1). This mathematical framework has broad applications in a number of domains, such as machine learning, signal processing, and medical imaging. For more information, see [9–11]. For example, zero points of MMOs can help reconstruct images from partial data in medical imaging. These operators are essential for creating optimization problems in machine learning that incorporate intricate models and huge data sets.

The study of solutions for (1) originated with Martinet’s pioneering work [12] in 1970, introducing the proximal point algorithm. Rockafellar [13] subsequently enhanced and extended this method, spawning a substantial body of literature that significantly advanced the field.

The proximal point approach has now undergone numerous modifications, each designed for a particular HS *Ω*. These improvements have been examined by numerous writers, who have presented a broad range of tactics meant to increase convergence rates and generalizable in various contexts [14–18].

The study of fixed points (FPs) for nonlinear mappings is a vibrant area within nonlinear analysis, driven by its diverse applications in fields like fuzzy logic, game theory, and inverse problems [19–22]. The estimation of common solutions for the zero points of MMOs and the FPs of nonlinear mappings has been the subject of a substantial amount of research. This area’s potential to address mathematical models with constraints, formulated as FP or zero-point problems [23–33], fuels ongoing investigation. The interplay between monotone operators and FP theory presents a fertile ground for further exploration.

Recent advancements in FP theory include Sahu et al.’s pioneering work [34], introducing the *S*-iteration technique for identifying common FPs of non-self quasi-nonexpansive mappings in uniformly convex Banach spaces. Their innovative approach, validated through numerical experiments, showcased tangible real-world applications. Building upon this success, Sahu et al. [35] further expanded the scope of FP methods by developing a variable anchoring iterative algorithm for solving problems on Hadamard manifolds, underscoring the pivotal role of geometric contexts in their analysis and demonstrating the versatility of FP theory in tackling complex mathematical problems.

## 2 Preliminaries

In this section, we recall a number of key terms and terminology that will be used in the sequel. Here, we assume that *ℜ* is a real HS, *M* is a non-empty, convex and closed (NCC) subset of *ℜ* ,  *fix*(*Q*) is the set of all FPs of the mapping *Q* ,   → ,  and  ⇀  is the strong and weak convergence of the sequence $\left\{\mu_{u}\right\}$ to *μ* ,  respectively.

**Definition 2.1.** [36] A *multi-valued mapping* $Q:ℜ\to 2^{ℜ}$ *is called*

(i) *Monotone, if*  ⟨ *ω* − *υ* , *μ* − *𝜐* ⟩ ≥ 0 ,  *provided that* *ω* ∈ *Q* ( *μ* )  *and* *υ* ∈ *Q* ( *𝜐* ) ; 
(ii) *Maximal monotone (MM), if the graph of* *Q* *not properly contained in the graph of any other monotone mapping and the mapping* *Q* *is monotone, where* graph(Q)={ (μ,υ)∈ℜ×ℜ:υ∈Q(μ)}.

The single-valued mapping related to the MM mapping *Q* is provided by $A_{\varphi }^{Q}={\left(I+\varphi Q\right)}^{-1}$ and it is known as the resolvent mapping of *Q*. The MM mapping *Q* has a mapping $A_{\varphi }^{Q}$, that is, firmly nonexpansive for each *φ* > 0 and fix$\left(A_{\varphi }^{Q}\right)=Q^{-1}(0)=\left\{\mu \in ℜ:0\in Q\mu \right\}.$ For more details; see [37].

**Definition 2.2.** [38] Let *M* be an NCC subset of *ℜ* .  We say that the mapping $P_{M}:ℜ\to M$ is a metric projection mapping if there is a unique point $P_{M}\mu \in M$ in order that


 ∥μ−PMμ∥= inf ⁡ ν∈M ∥μ−ν∥, for all μ∈ℜ.

Also, for all *μ* ∈ *ℜ* ,  *ν* ∈ *M* ,  we have


$$\langle \mu -P_{M}\mu ,P_{M}\mu -\nu \rangle \geq 0.$$

**Definition 2.3.** [39] A mapping *Q* : *ℜ* → *ℜ* with Fix (Q)≠∅ is called

(a) *ς* − DMM, where *ς* is a real number with ς∈ (−∞,1) if


$$\langle \mu -\mu^{*},\mu -Q\mu \rangle \geq \frac{1-\varsigma }{2}{∥\mu -Q\mu ∥}^{2},$$

for all *μ* ∈ *ℜ* and $\mu^{*}\in Fix\left(Q\right).$

(b) *ϕ*-generalized DMM, where *ϕ* ∈ *ℝ* − { 0 }  if


$$\phi \langle \mu -\mu^{*},\mu -Q\mu \rangle \geq {∥\mu -Q\mu ∥}^{2},$$

for all *μ* ∈ *ℜ* and $\mu^{*}\in Fix\left(Q\right).$

**Remark 2.4.** [40] Based on the above definition, the *ϕ*-generalized DMM is $(1-\frac{2}{\phi })-$DMM, for *ϕ* > 0 . 

**Lemma 2.5.** [37] The following relations hold : 

(i) ${∥\omega_{1}\varkappa_{1}+(1-\omega_{1})\varkappa_{2}∥}^{2}=\omega_{1}{∥\varkappa_{1}∥}^{2}+(1-\omega_{1}){∥\varkappa_{2}∥}^{2}-\omega_{1}(1-\omega_{1}){∥\varkappa_{1}-\varkappa_{2}∥}^{2};$
(ii) ${∥\varkappa_{1}+\varkappa_{2}∥}^{2}\leq {∥\varkappa_{1}∥}^{2}+2\langle \varkappa_{1},\varkappa_{1}+\varkappa_{2}\rangle ;$
(iii) ${∥\varkappa_{1}\pm \varkappa_{2}∥}^{2}={∥\varkappa_{1}∥}^{2}\pm 2\langle \varkappa_{1},\varkappa_{2}\rangle +{∥\varkappa_{2}∥}^{2};$
(iv) ${∥\omega_{1}\varkappa_{1}+\omega_{2}\varkappa_{2}+\omega_{3}\varkappa_{3}∥}^{2}=\omega_{1}{∥\varkappa_{1}∥}^{2}+\omega_{2}{∥\varkappa_{2}∥}^{2}+\omega_{3}{∥\varkappa_{3}∥}^{2}-\omega_{1}\omega_{2}∥\varkappa_{1}-\varkappa_{2}∥-\omega_{1}\omega_{3}∥\varkappa_{1}-\varkappa_{3}∥-\omega_{2}\omega_{3}∥\varkappa_{2}-\varkappa_{3}∥,$

for $\varkappa_{1},\varkappa_{2},\varkappa_{3}\in ℜ$ and $\omega_{1},\omega_{2},\omega_{3}\in [0,1]$ with $\omega_{1}+\omega_{2}+\omega_{3}=1.$

**Lemma 2.6.** [38] Let the mapping *Q* be a nonexpansive on *ℜ*. If a sequence $\{\mu_{u}\}⇀\mu^{*}\in ℜ$ and $\{\mu_{u}-Q\mu_{u}\}\to 0,$ then $\mu^{*}\in fix(Q),$ that is, *Q* is demiclosed on *ℜ* . 

**Lemma 2.7.** [41] Assume that *Q* : *M* → *ℜ* is a *ϕ* − DMM in order that *fix* ( *Q* ) ≠ *∅* and *ϕ* ∈ ( − *∞* , 1 ) .  Also, let *b* ∈ ( 0 , *∞* )  and define *V* = ( 1 − *b* ) *I* + *bQ* .  Then, the following axioms are true:

(1) if *b* ≠ 0 ,  *fix* ( *Q* ) = *fix* ( *V* ) , 
(2) *fix*(*Q*) is a closed convex subset of *ℜ* , 
(3) for *b* ∈ ( 0 , 1 − *ϕ* ) ,  the mapping *V* is a quasi-nonexpansive.

**Lemma 2.8.** [42] The *fix*(*Q*) is closed and convex, provided that *Q* is a *ϕ*-generalized DMM on *ℜ* ,  where *ϕ* ∈ *ℝ* − { 0 } . 

**Lemma 2.9.** [43] Assume that $\left\{\mu_{u}\right\}$ is a sequence of non-negative real numbers. Let


$$\mu_{u+1}\leq (1-q_{u})\mu_{u}+q_{u}r_{u},\text{ for all }u\geq 1,$$

where $\left\{q_{u}\right\}\subseteq [0,1],$ $\left\{r_{u}\right\}\subseteq \mathbb{R}$ such that $\sum_{u=0}^{\infty }q_{u}=\infty$ and limsup ⁡ u→∞ru≤0, then lim ⁡ u→∞μu=0.

**Lemma 2.10.** [41] Assume that *M* is a nonempty convex subset of the HS *ℜ* ,  and ${\left\{Q_{j}\right\}}_{j=1}^{\infty }:M\to ℜ$ is an infinite family of $b_{j}-$DMMs with $\sup\{b_{j}:j\in \mathbb{N}\}<1$ and $\cap_{j=1}^{\infty }fix(Q_{j})\neq \emptyset .$ Let ${\left\{\kappa_{j}\right\}}_{j=1}^{\infty }$ be a positive sequence such that $\sum_{j=1}^{\infty }\kappa_{j}=\infty .$ Then, $\sum_{j=1}^{\infty }\kappa_{j}Q_{j}:M\to ℜ$ is a *b*–DMM with $b=\sup\{b_{j}:j\in \mathbb{N}\}<1$ and $fix\left(\sum_{j=1}^{\infty }\kappa_{j}Q_{j}\right)=\cap_{j=1}^{\infty }Fix(Q_{j}).$

## 3 Strong convergence results

This section contains the definition of our algorithm and proof of the suggested algorithm’s strong convergence. In this part, we assume that $Q_{j}:ℜ\to ℜ$ is a finite class of $\phi_{j}$-generalized DMMs where $\phi_{j}\in (0,\infty )$ and $I-\phi_{j}$ is demiclosed at the origin for all *j* = 1 , 2 , ⋯ , *u* .  Further, *g* : *ℜ* → *ℜ* is a $b_{1}$-contraction mapping with $b_{1}\in [0,1)$ and $B_{k}:ℜ\to 2^{ℜ}$ is a finite class of multi-valued monotone mappings for every *k* = 1 , 2 , ⋯ , *P* . 

Algorithms for DMM play a crucial role in approximating FPs, offering efficient and robust methods for solving complex optimization problems. By leveraging DMMs, these algorithms ensure convergence to fixed points in various mathematical structures, including HSs. Their significance extends to numerous applications, such as variational inequalities, split feasibility problems, and minimization problems, facilitating solutions in fields like machine learning, data analysis, and control systems. So, our algorithm here is as follows:

**Algorithm 3.1.** *Assume that* $\mu_{1},\mu_{2}\in ℜ.$ *Compute* $\{\alpha_{u}\},$ $\{\beta_{u}\},$ $\{\gamma_{u}\},$ $\left\{\ell_{u}\right\}$ *and* $\left\{\mu_{u}\right\}$ *by*


$$\begin{array}{ccc} \{\begin{array}{c} \alpha_{u}=\mu_{u}+\varrho_{u}\left(\mu_{u}-\mu_{u-1}\right),\mathrm{where}\varrho_{u}=\{\begin{array}{c} \min\left\{\frac{\tau_{u}}{∥\mu_{u}-\mu_{u-1}∥}\right\},\text{ if }\mu_{u}\neq \mu_{u-1},\\ 0,\mathrm{otherwise,} \end{array}\\ \beta_{u}=A_{\varphi_{P}}^{B_{P}}A_{\varphi_{P-1}}^{B_{P-1}}\cdots A_{\varphi_{1}}^{B_{1}}\alpha_{u},\\ \gamma_{u}=(1-\lambda )\beta_{u}+\lambda \left(\overset{u}{\underset{j=1}{\sum }}{𝜗}_{j}\left[\left(1-b\right)I+bQ_{j}\right]\beta_{u}\right),\\ \ell_{u}=\rho \left(\overset{u}{\underset{j=1}{\sum }}{𝜗}_{j}\left[\left(1-b\right)I+bQ_{j}\right]\beta_{u}\right)\gamma_{u},\\ \mu_{u+1}=\delta_{u}g\left(\mu_{u}\right)+(1-\delta_{u})\ell_{u}. \end{array} & & \end{array}$$ (2)

**Lemma 3.2.** *Let* $\phi_{j}\in (0,\infty ),$ *and* $V_{j}:ℜ\to ℜ$ *be a self-mapping defined by* $V_{j}=(1-b)I+bQ_{j},$ *for all* *j* = 1 , 2 , ⋯ , *u* *with* $b\in (0,\lambda_{1})$ *and* $\lambda_{1}\in (0,1).$ *Then*, $V_{j}$ is $\phi_{j}b$-*generalized DMM if* $\phi_{j}:ℜ\to ℜ$ is $\phi_{j}$-*generalized DMM for all* *j* = 1 , 2 , ⋯ , *u* . 

**Proof.** Let $\mu^{*}\in fix\left(V_{j}\right)=fix\left(Q_{j}\right),$ *j* = 1 , 2 , ⋯ , *u* and by the defintion of $\phi_{j}$-generalized DMM, we have


$$\begin{array}{cccc} \left\langle \mu -\mu^{*},\mu -V_{j}\mu \right\rangle & = & \left\langle \mu -\mu^{*},\mu -\left[(1-b)I+bQ_{j}\right]\mu \right\rangle & \\ & = & \left\langle \mu -\mu^{*},b\left(\mu -Q_{j}\mu \right)\right\rangle & \\ & = & b\langle \mu -\mu^{*},\mu -Q_{j}\mu \rangle & \\ & \geq & \frac{b}{\phi_{j}}{∥\mu -Q_{j}\mu ∥}^{2}, \end{array}$$ (3)

for all *μ* ∈ *ℜ* .  Since


$$\begin{array}{rcll} \mu -Q_{j}\mu & = & \mu -\frac{\mu }{b}\left[V_{j}-(1-b)I\right] & \\ & = & \mu -\frac{\mu }{b}V_{j}+\frac{\mu }{b}-\mu & \\ & = & \frac{\mu }{b}-\frac{\mu }{b}V_{j} & \\ & = & \frac{1}{b}\left(\mu -V_{j}\mu \right). & \end{array}$$

Then,


$$\begin{array}{ccc} {∥\mu -Q_{j}\mu ∥}^{2}=\frac{1}{b^{2}}{∥\mu -V_{j}\mu ∥}^{2}. & & \end{array}$$ (4)

From (4) in (3), we get$\frac{1}{b\phi_{j}}{∥\mu -V_{j}\mu ∥}^{2}$


$$\begin{array}{rcll} \left\langle \mu -\mu^{*},\mu -V_{j}\mu \right\rangle & \geq & \frac{b}{\phi_{j}}{∥\mu -Q_{j}\mu ∥}^{2} & \\ & = & \frac{b}{\phi_{j}}\frac{1}{b^{2}}{∥\mu -V_{j}\mu ∥}^{2} & \\ & = & \frac{1}{b\phi_{j}}{∥\mu -V_{j}\mu ∥}^{2}. & \end{array}$$

Thus, one can write


$$b\phi_{j}\langle \mu -\mu^{*},\mu -V_{j}\mu \rangle \geq {∥\mu -V_{j}\mu ∥}^{2}.$$

This proves that $V_{j}$ is $\phi_{j}b$-generalized demimetric. □

**Lemma 3.3.** Assume that $\left\{\mu_{u}\right\}$ is a bounded sequence in *ℝ* and $\mu^{*}\in \Theta =\left\{\cap_{j=1}^{u}fix(Q_{j})\right\}\cap \left\{\cap_{k=1}^{P}B_{j}^{-1}(0)\right\}\neq \emptyset .$ Define $\Phi^{P}=A_{\varphi_{P}}^{B_{P}}A_{\varphi_{P-1}}^{B_{P-1}}\cdots A_{\varphi_{1}}^{B_{1}},$ where $A_{\varphi_{k}}^{B_{k}}$ refer to the resolvent mapping of $B_{k}$ for all *k* = 1 , 2 , ⋯ , *P* .  If


$$\{\begin{array}{c} ∥\Phi^{k-1}\alpha_{u}-\Phi^{k-1}\alpha_{u}∥\to 0,\\ ∥\mu_{u}-\alpha_{u}∥\to 0,\\ ∥\mu_{u}-\beta_{u}∥\to 0,\\ ∥\beta_{u}-Z\beta_{u}∥\to 0, \end{array}\text{ as }u\to \infty .$$

Then, limsup ⁡ u→∞⟨g(μ∗)−μ∗,μu−μ∗⟩≤0.

**Proof.** Since $\left\{\mu_{u}\right\}$ is bounded, then there exists a subsequence $\left\{\mu_{u_{b}}\right\}$ of $\left\{\mu_{u}\right\}$ such that $\mu_{u_{b}}⇀\mu^{\prime }\in M$ and


limsup ⁡ u→∞⟨g(μ∗)−μ∗,μu−μ∗⟩=limsup ⁡ u→∞⟨g(μ∗)−μ∗,μub−μ∗⟩=⟨g(μ∗)−μ∗,μ′−μ∗⟩. (5)

By Lemma 3.2, $V_{j}$ is an $\phi_{j}b$-generalized DMM, provided that $\phi_{j}:ℜ\to ℜ$ is $\phi_{j}$-generalized DMM for all $b\in (0,\lambda_{1})$ with $\lambda_{1}\in (0,1)$ for each *j* = 1 , 2 , ⋯ , *u* .  Utilizing Remark 2.4, we have $V_{j}$ is $\left(1-\frac{2}{\phi_{j}b}\right)$-demimetric. Thanks to Lemma 2.10, $Z=\sum_{j=1}^{u}{𝜗}_{j}V_{j}$ is DMM and so nonexpansive. Furthermore, because $∥\beta_{u_{b}}-Z\beta_{u_{b}}∥\to 0,$ $∥\beta_{u_{b}}-\mu_{u_{b}}∥\to 0$ as *b* → *∞* and $\mu_{u_{b}}\to \mu^{\prime },$ then, by Lemmas 2.6, 2.7, and 2.10, one can obtain


$$\mu^{\prime }\in fix(Z)=\cap_{j=1}^{u}fix(Q_{j})=\cap_{j=1}^{u}fix(Q_{j}).$$

Next, we show that $\mu^{\prime }\in \cap_{k=1}^{P}B_{j}^{-1}(0).$ According to given assumption, we get


lim ⁡ u→∞ ∥Φk−1αu−Φkαu∥=0 for all k.

By induction, if *k* = 1, we have lim ⁡ u→∞ ∥Φ0αu−Φ1αu∥=0 or we can write


lim ⁡ u→∞ ∥αu−Aφ1B1αu∥=0 and lim ⁡ u→∞ ∥αu−μu∥=0.

Hence, one has


lim ⁡ u→∞ ∥μu−Aφ1B1μu∥=0.

Analogously, if *k* = 2, we have lim ⁡ u→∞ ∥Φ1αu−Φ2αu∥=0. Now, we assume that


$$\begin{array}{cccc} ∥A_{\varphi_{2}}^{B_{2}}\alpha_{u}-\alpha_{u}∥ & = & ∥A_{\varphi_{2}}^{B_{2}}\alpha_{u}-A_{\varphi_{2}}^{B_{2}}A_{\varphi_{1}}^{B_{1}}\alpha_{u}+A_{\varphi_{2}}^{B_{2}}A_{\varphi_{1}}^{B_{1}}\alpha_{u}-\alpha_{u}∥ & \\ & \leq & ∥A_{\varphi_{2}}^{B_{2}}\alpha_{u}-A_{\varphi_{2}}^{B_{2}}A_{\varphi_{1}}^{B_{1}}\alpha_{u}∥+∥A_{\varphi_{2}}^{B_{2}}A_{\varphi_{1}}^{B_{1}}\alpha_{u}-\alpha_{u}∥ & \\ & \leq & ∥\alpha_{u}-A_{\varphi_{1}}^{B_{1}}\alpha_{u}∥+∥A_{\varphi_{2}}^{B_{2}}A_{\varphi_{1}}^{B_{1}}\alpha_{u}-\alpha_{u}∥, \end{array}$$ (6)

and


$$\begin{array}{cccc} ∥A_{\varphi_{2}}^{B_{2}}A_{\varphi_{1}}^{B_{1}}\alpha_{u}-\alpha_{u}∥ & = & ∥A_{\varphi_{2}}^{B_{2}}A_{\varphi_{1}}^{B_{1}}\alpha_{u}-A_{\varphi_{1}}^{B_{1}}\alpha_{u}+A_{\varphi_{1}}^{B_{1}}\alpha_{u}-\alpha_{u}∥ & \\ & \leq & ∥A_{\varphi_{2}}^{B_{2}}A_{\varphi_{1}}^{B_{1}}\alpha_{u}-A_{\varphi_{1}}^{B_{1}}\alpha_{u}∥+∥A_{\varphi_{1}}^{B_{1}}\alpha_{u}-\alpha_{u}∥. \end{array}$$ (7)

Taking *u* → *∞* in (7), we have


lim ⁡ u→∞ ∥Aφ2B2Aφ1B1αu−αu∥=0. (8)

From (8) in (6) and taking *u* → *∞* ,  we can write


lim ⁡ u→∞ ∥Aφ2B2αu−αu∥=0.

Hence,


lim ⁡ u→∞ ∥Aφ2B2μu−μu∥=0.

Following the same approach, we have


lim ⁡ u→∞ ∥AφkBkαu−αu∥=0 for all k=1,2,⋯,P,

which yields, $\mu^{\prime }\in fix\left(A_{\varphi_{k}}^{B_{k}}\right).$ Thus, $\mu^{\prime }\in B_{k}^{-1}(0)$ for all *k* = 1 , 2 , ⋯ , *P* .  This proves that $\mu^{\prime }\in \cap_{j=1}^{u}B_{k}^{-1}(0).$ Therefore, $\mu^{\prime }\in \Theta .$ So, by the equality (5) and metric projection properties, we have the result. □

**Theorem 3.4.** Let the solution set $\Theta =\left\{\cap_{j=1}^{u}fix(Q_{j})\right\}\cap \left\{\cap_{k=1}^{P}B_{j}^{-1}(0)\right\}\neq \emptyset$ and $\left\{\mu_{u}\right\}$ be a sequence given by Algorithm 3.1 for any $\mu_{1}\in ℜ,$ where *b* ∈ ( 0 , *ρ* )  with *ρ* ∈ ( 0 , 1 ) ,  *ϱ* > 0 ,  $\phi_{j}b>0,$ and $\{\delta_{u}\},\{{𝜗}_{u}\},\{\lambda \}\in (0,1),$ $\sum_{j=1}^{\infty }\mu_{j}=1$ under the following hypotheses:

(h_1_) $\sum_{j=1}^{\infty }\delta_{u}=\infty ,$ lim ⁡ u→∞δu=0,
(h_2_) for a positive term sequence $\left\{\tau_{u}\right\}$, lim ⁡ u→∞τuδu=0.

Then $\mu_{u}\to \mu^{*}\in P_{\Theta }g(\mu^{*})\in \Theta .$

**Proof.** Consider $V_{j}=(1-b)I+bQ_{j},$ and $Z=\sum_{j=1}^{u}{𝜗}_{j}V_{j}$. The algorithm 3.1 can be expressed as


$$\begin{array}{ccc} \{\begin{array}{c} \alpha_{u}=\mu_{u}+\varrho_{u}\left(\mu_{u}-\mu_{u-1}\right),\text{ where }\varrho_{u}=\{\begin{array}{c} \min\left\{\frac{\tau_{u}}{∥\mu_{u}-\mu_{u-1}∥}\right\},\text{ if }\mu_{u}\neq \mu_{u-1},\\ 0,\text{ otherwise,} \end{array}\\ \beta_{u}=A_{\varphi_{P}}^{B_{P}}A_{\varphi_{P-1}}^{B_{P-1}}\cdots A_{\varphi_{1}}^{B_{1}}\alpha_{u},\\ \gamma_{u}=(1-\lambda )\beta_{u}+\lambda Z\beta_{u},\\ \ell_{u}=\rho Z\gamma_{u},\\ \mu_{u+1}=\delta_{u}g\left(\mu_{u}\right)+(1-\delta_{u})\ell_{u}. \end{array} & & \end{array}$$ (9)

As $A_{\varphi_{k}}^{B_{k}}$ is firmly nonexpansive, then, it is nonexpansive for all *k* = 1 , 2 , ⋯ , *P* .  Thus, $fix\left(A_{\varphi_{k}}^{B_{k}}\right)$ is closed and convex for all *k* = 1 , 2 , ⋯ , *P* .  Moreover, $Q_{j}$ is $\phi_{j}-$DMM, so by Lemma 2.8, we get, for all *k* = 1 , 2 , ⋯ , *P* ,  $fix(Q_{j})$ is closed and convex. This implies that *Θ* is NCC. Thus, $P_{\Theta }$ is well-defined. From the definition of $\left\{\varrho_{u}\right\}$ and the hypotheses $(h_{2}),$ we conclude that lim ⁡ u→∞ϱuδu ∥μu−μu−1∥=0 (Remark 3.1 [43]).

We claim that $\left\{\mu_{u}\right\}$ is bounded. Assume that $\Phi^{P}=A_{\varphi_{P}}^{B_{P}}A_{\varphi_{P-1}}^{B_{P-1}}\cdots A_{\varphi_{1}}^{B_{1}}$ and $\mu^{*}\in \Theta ,$ we have


$$\begin{array}{cccc} ∥\alpha_{u}-\mu^{*}∥ & = & ∥\mu_{u}+\varrho_{u}\left(\mu_{u}-\mu_{u-1}\right)-\mu^{*}∥ & \\ & \leq & ∥\mu_{u}-\mu^{*}∥+\varrho_{u}∥\mu_{u}-\mu_{u-1}∥, \end{array}$$ (10)

$$\begin{array}{cccc} ∥\beta_{u}-\mu^{*}∥ & = & ∥A_{\varphi_{P}}^{B_{P}}A_{\varphi_{P-1}}^{B_{P-1}}\cdots A_{\varphi_{1}}^{B_{1}}\alpha_{u}-\mu^{*}∥ & \\ & = & ∥A_{\varphi_{P}}^{B_{P}}\Phi^{P-1}\alpha_{u}-\mu^{*}∥ & \\ & \leq & ∥\Phi^{P-1}\alpha_{u}-\mu^{*}∥ & \\ & \leq & ∥\alpha_{u}-\mu^{*}∥, \end{array}$$ (11)

$$\begin{array}{cccc} ∥\gamma_{u}-\mu^{*}∥ & = & ∥(1-\lambda )\beta_{u}+\lambda Z\beta_{u}-\mu^{*}∥ & \\ & = & ∥(1-\lambda )\left(\beta_{u}-\mu^{*}\right)+\lambda \left(Z\beta_{u}-\mu^{*}\right)∥ & \\ & \leq & (1-\lambda )∥\beta_{u}-\mu^{*}∥+\lambda ∥Z\beta_{u}-\mu^{*}∥ & \\ & \leq & ∥\beta_{u}-\mu^{*}∥, \end{array}$$ (12)

and


$$\begin{array}{cccc} ∥\ell_{u}-\mu^{*}∥ & = & ∥\rho Z\gamma_{u}-\mu^{*}∥ & \\ & \leq & ∥Z\gamma_{u}-\mu^{*}∥\text{ (since }\rho <1) & \\ & \leq & ∥\gamma_{u}-\mu^{*}∥. \end{array}$$ (13)

Since lim ⁡ u→∞ϱuδu ∥μu−μu−1∥=0, then there exists *N* > 0 such that $\frac{\varrho_{u}}{\delta_{u}}∥\mu_{u}-\mu_{u-1}∥\leq N.$ Utilizing the inequalities (10)-(13), we can write


$$\begin{array}{rcll} ∥\mu_{u+1}-\mu^{*}∥ & = & ∥\delta_{u}g\left(\mu_{u}\right)+(1-\delta_{u})\ell_{u}-\mu^{*}∥ & \\ & \leq & \delta_{u}∥g\left(\mu_{u}\right)-\mu^{*}∥+(1-\delta_{u})∥\ell_{u}-\mu^{*}∥ & \\ & \leq & \delta_{u}∥g\left(\mu_{u}\right)-\mu^{*}∥+(1-\delta_{u})∥\gamma_{u}-\mu^{*}∥ & \\ & \leq & \delta_{u}∥g\left(\mu_{u}\right)-g(\mu^{*})∥+\delta_{u}∥g(\mu^{*})-\mu^{*})∥+(1-\delta_{u})∥\beta_{u}-\mu^{*}∥ & \\ & \leq & \delta_{u}b_{1}∥\mu_{u}-\mu^{*}∥+\delta_{u}∥g(\mu^{*})-\mu^{*})∥+(1-\delta_{u})∥\alpha_{u}-\mu^{*}∥ & \\ & \leq & \delta_{u}b_{1}∥\mu_{u}-\mu^{*}∥+\delta_{u}∥g(\mu^{*})-\mu^{*})∥+(1-\delta_{u})\left[∥\mu_{u}-\mu^{*}∥+\varrho_{u}∥\mu_{u}-\mu_{u-1}∥\right] & \\ & \leq & \left(1-\delta_{u}(1-b_{1})\right)∥\mu_{u}-\mu^{*}∥+\delta_{u}∥g(\mu^{*})-\mu^{*})∥+\delta_{u}\frac{\varrho_{u}}{\delta_{u}}∥\mu_{u}-\mu_{u-1}∥ & \\ & \leq & \left(1-\delta_{u}(1-b_{1})\right)∥\mu_{u}-\mu^{*}∥+\delta_{u}\left(∥g(\mu^{*})-\mu^{*})∥+N\right) & \\ & \leq & \left(1-\delta_{u}(1-b_{1})\right)∥\mu_{u}-\mu^{*}∥+\delta_{u}(1-b_{1})\left(\frac{1}{1-b_{1}}\left[∥g(\mu^{*})-\mu^{*})∥+N\right]\right) & \\ & \leq & \max\left\{∥\mu_{u}-\mu^{*}∥,\frac{∥g(\mu^{*})-\mu^{*})∥+N}{1-b_{1}}\right\}, & \end{array}$$

which yields, the sequence $\left\{\mu_{u}\right\}$ is bounded and hence the sequences $\{\alpha_{u}\},$ $\{\beta_{u}\},$ $\{\gamma_{u}\},$ $\left\{\ell_{u}\right\}$ are also bounded.

Now,


$$\begin{array}{cccc} {∥\alpha_{u}-\mu^{*}∥}^{2} & = & {∥\mu_{u}+\varrho_{u}\left(\mu_{u}-\mu_{u-1}\right)-\mu^{*}∥}^{2} & \\ & = & {∥\mu_{u}-\mu^{*}∥}^{2}+\varrho_{u}^{2}{∥\mu_{u}-\mu_{u-1}∥}^{2}+2\varrho_{u}∥\mu_{u}-\mu^{*}∥∥\mu_{u}-\mu_{u-1}∥, \end{array}$$ (14)

$$\begin{array}{cccc} {∥\beta_{u}-\mu^{*}∥}^{2} & = & {∥A_{\varphi_{P}}^{B_{P}}A_{\varphi_{P-1}}^{B_{P-1}}\cdots A_{\varphi_{1}}^{B_{1}}\alpha_{u}-\mu^{*}∥}^{2} & \\ & = & {∥A_{\varphi_{P}}^{B_{P}}\Phi^{P-1}\alpha_{u}-\mu^{*}∥}^{2} & \\ & = & {∥\Phi^{P-1}\alpha_{u}-\mu^{*}∥}^{2}\\ & \leq & {∥\alpha_{u}-\mu^{*}∥}^{2}. & \end{array}$$ (15)

By Lemma 2.5, we can write


$$\begin{array}{rcll} {∥\gamma_{u}-\mu^{*}∥}^{2} & = & {∥(1-\lambda )\beta_{u}+\lambda Z\beta_{u}-\mu^{*}∥}^{2} & \\ & = & {∥(1-\lambda )\left(\beta_{u}-\mu^{*}\right)+\lambda \left(Z\beta_{u}-\mu^{*}\right)∥}^{2} & \\ & \leq & (1-\lambda ){∥\beta_{u}-\mu^{*}∥}^{2}+\lambda {∥Z\beta_{u}-\mu^{*}∥}^{2}-\lambda (1-\lambda ){∥\beta_{u}-Z\beta_{u}∥}^{2} & \\ & \leq & (1-\lambda ){∥\beta_{u}-\mu^{*}∥}^{2}+\lambda {∥\beta_{u}-\mu^{*}∥}^{2} & \\ & = & {∥\beta_{u}-\mu^{*}∥}^{2}, & \end{array}$$

hence,


$$\begin{array}{ccc} {∥\gamma_{u}-\mu^{*}∥}^{2}\leq {∥\beta_{u}-\mu^{*}∥}^{2}. & & \end{array}$$ (16)

Also,


$$\begin{array}{cccc} {∥\ell_{u}-\mu^{*}∥}^{2} & = & {∥\rho Z\gamma_{u}-\mu^{*}∥}^{2} & \\ & \leq & {∥Z\gamma_{u}-\mu^{*}∥}^{2} & \\ & \leq & {∥\gamma_{u}-\mu^{*}∥}^{2}. \end{array}$$ (17)

From the inequalities (14)–(17) and Lemma 2.5, we get


$$\begin{array}{cccc} & & {∥\mu_{u+1}-\mu^{*}∥}^{2} & \\ & = & {∥\delta_{u}g\left(\mu_{u}\right)+(1-\delta_{u})\ell_{u}-\mu^{*}∥}^{2} & \\ & = & {∥\delta_{u}\left(g\left(\mu_{u}\right)-\mu^{*}\right)+(1-\delta_{u})\left(\ell_{u}-\mu^{*}\right)∥}^{2} & \\ & = & {∥\delta_{u}\left(g\left(\mu_{u}\right)-g(\mu^{*})\right)+\delta_{u}\left(g(\mu^{*})-\mu^{*}\right)+(1-\delta_{u})\left(\ell_{u}-\mu^{*}\right)∥}^{2} & \\ & = & {∥\delta_{u}\left(g\left(\mu_{u}\right)-g(\mu^{*})\right)+(1-\delta_{u})\left(\ell_{u}-\mu^{*}\right)∥}^{2}+2\langle \delta_{u}\left(g\left(\mu_{u}\right)-\mu^{*}\right),\mu_{u+1}-\mu^{*}\rangle & \\ & \leq & \delta_{u}{∥g\left(\mu_{u}\right)-g(\mu^{*})∥}^{2}+(1-\delta_{u}){∥\ell_{u}-\mu^{*}∥}^{2}+2\delta_{u}\langle g\left(\mu^{*}\right)-\mu^{*},\mu_{u+1}-\mu^{*}\rangle & \\ & \leq & \delta_{u}b_{1}{∥\mu_{u}-\mu^{*}∥}^{2}+(1-\delta_{u}){∥\ell_{u}-\mu^{*}∥}^{2}+2\delta_{u}\langle g\left(\mu^{*}\right)-\mu^{*},\mu_{u+1}-\mu^{*}\rangle & \\ & \leq & \delta_{u}b_{1}{∥\mu_{u}-\mu^{*}∥}^{2}+(1-\delta_{u}){∥\gamma_{u}-\mu^{*}∥}^{2}+2\delta_{u}\langle g\left(\mu^{*}\right)-\mu^{*},\mu_{u+1}-\mu^{*}\rangle , \end{array}$$ (18)

which implies that


$$\begin{array}{cccc} & & {∥\mu_{u+1}-\mu^{*}∥}^{2} & \\ & \leq & \delta_{u}b_{1}{∥\mu_{u}-\mu^{*}∥}^{2}+(1-\delta_{u}){∥\beta_{u}-\mu^{*}∥}^{2}+2\delta_{u}\langle g\left(\mu^{*}\right)-\mu^{*},\mu_{u+1}-\mu^{*}\rangle & \\ & \leq & \delta_{u}b_{1}{∥\mu_{u}-\mu^{*}∥}^{2}+(1-\delta_{u}){∥\alpha_{u}-\mu^{*}∥}^{2}+2\delta_{u}\langle g\left(\mu^{*}\right)-\mu^{*},\mu_{u+1}-\mu^{*}\rangle & \\ & \leq & \delta_{u}b_{1}{∥\mu_{u}-\mu^{*}∥}^{2} & \\ & & +(1-\delta_{u})\left[{∥\mu_{u}-\mu^{*}∥}^{2}+\varrho_{u}^{2}{∥\mu_{u}-\mu_{u-1}∥}^{2}+2\varrho_{u}∥\mu_{u}-\mu^{*}∥∥\mu_{u}-\mu_{u-1}∥\right] & \\ & & +2\delta_{u}\langle g\left(\mu^{*}\right)-\mu^{*},\mu_{u+1}-\mu^{*}\rangle & \\ & \leq & \left[1-\delta_{u}(1-b_{1})\right]{∥\mu_{u}-\mu^{*}∥}^{2}+\varrho_{u}^{2}{∥\mu_{u}-\mu_{u-1}∥}^{2}+2\varrho_{u}∥\mu_{u}-\mu^{*}∥∥\mu_{u}-\mu_{u-1}∥ & \\ & & +2\delta_{u}\langle g\left(\mu^{*}\right)-\mu^{*},\mu_{u+1}-\mu^{*}\rangle & \\ & \leq & \left[1-\delta_{u}(1-b_{1})\right]{∥\mu_{u}-\mu^{*}∥}^{2}+\delta_{u}(1-b_{1})\{\frac{2}{1-b_{1}}\langle g\left(\mu^{*}\right)-\mu^{*},\mu_{u+1}-\mu^{*}\rangle & \\ & & +\frac{\varrho_{u}^{2}}{\delta_{u}\left(1-b_{1}\right)}{∥\mu_{u}-\mu_{u-1}∥}^{2}+\frac{2\varrho_{u}}{\delta_{u}\left(1-b_{1}\right)}∥\mu_{u}-\mu^{*}∥∥\mu_{u}-\mu_{u-1}∥\}. \end{array}$$ (19)

Inequality (19) can be simplified as


$$\begin{array}{ccc} t_{u+1}\leq (1-q_{u})t_{u}+q_{u}r_{u}, & & \end{array}$$ (20)

where


$$\begin{array}{rcll} t_{u} & = & {∥\mu_{u}-\mu^{*}∥}^{2}, & \\ q_{u} & = & \delta_{u}(1-b_{1}), & \\ r_{u} & = & \frac{2}{1-b_{1}}\langle g\left(\mu^{*}\right)-\mu^{*},\mu_{u+1}-\mu^{*}\rangle +\frac{\varrho_{u}^{2}}{\delta_{u}\left(1-b_{1}\right)}{∥\mu_{u}-\mu_{u-1}∥}^{2} & \\ & & +\frac{2\varrho_{u}}{\delta_{u}\left(1-b_{1}\right)}∥\mu_{u}-\mu^{*}∥∥\mu_{u}-\mu_{u-1}∥. & \end{array}$$

The rest of the proof will be divided into the following two cases:

Case 1: For some $u\geq u_{0}$, where $u_{0}\in \mathbb{N}$, assume that $\{t_{u}\}\subseteq \mathbb{R}$ is a nonincreasing sequence. Then the limit exists because the sequence $\left\{t_{u}\right\}$ is monotonic and bounded. Since $b_{1}<1$ and using (18), we get


$$\begin{array}{rcll} & & {∥\mu_{u+1}-\mu^{*}∥}^{2} & \\ & \leq & \delta_{u}b_{1}{∥\mu_{u}-\mu^{*}∥}^{2}+(1-\delta_{u}){∥\gamma_{u}-\mu^{*}∥}^{2}+2\delta_{u}\langle g\left(\mu^{*}\right)-\mu^{*},\mu_{u+1}-\mu^{*}\rangle & \\ & \leq & \delta_{u}b_{1}{∥\mu_{u}-\mu^{*}∥}^{2}+(1-\delta_{u})\left[{∥(1-\lambda )\left(\beta_{u}-\mu^{*}\right)+\lambda \left(Z\beta_{u}-\mu^{*}\right)∥}^{2}\right] & \\ & & +2\delta_{u}\langle g\left(\mu^{*}\right)-\mu^{*},\mu_{u+1}-\mu^{*}\rangle & \\ & \leq & \delta_{u}b_{1}{∥\mu_{u}-\mu^{*}∥}^{2} & \\ & & +(1-\delta_{u})\left[(1-\lambda ){∥\beta_{u}-\mu^{*}∥}^{2}+\lambda {∥Z\beta_{u}-\mu^{*}∥}^{2}-\lambda (1-\lambda ){∥\beta_{u}-Z\beta_{u}∥}^{2}\right] & \\ & & +2\delta_{u}\langle g\left(\mu^{*}\right)-\mu^{*},\mu_{u+1}-\mu^{*}\rangle & \\ & = & \delta_{u}{∥\mu_{u}-\mu^{*}∥}^{2}+(1-\delta_{u})\left[{∥\beta_{u}-\mu^{*}∥}^{2}-\lambda (1-\lambda ){∥\beta_{u}-Z\beta_{u}∥}^{2}\right] & \\ & & +2\delta_{u}\langle g\left(\mu^{*}\right)-\mu^{*},\mu_{u+1}-\mu^{*}\rangle & \\ & \leq & \delta_{u}{∥\mu_{u}-\mu^{*}∥}^{2}+(1-\delta_{u})\left[{∥\alpha_{u}-\mu^{*}∥}^{2}-\lambda (1-\lambda ){∥\beta_{u}-Z\beta_{u}∥}^{2}\right] & \\ & & +2\delta_{u}\langle g\left(\mu^{*}\right)-\mu^{*},\mu_{u+1}-\mu^{*}\rangle & \\ & \leq & \delta_{u}{∥\mu_{u}-\mu^{*}∥}^{2}+ & \\ & & (1-\delta_{u})[{∥\mu_{u}-\mu^{*}∥}^{2}+\varrho_{u}^{2}{∥\mu_{u}-\mu_{u-1}∥}^{2}+2\varrho_{u}∥\mu_{u}-\mu^{*}∥∥\mu_{u}-\mu_{u-1}∥ & \\ & & -\lambda (1-\lambda ){∥\beta_{u}-Z\beta_{u}∥}^{2}]+2\delta_{u}\langle g\left(\mu^{*}\right)-\mu^{*},\mu_{u+1}-\mu^{*}\rangle & \\ & \leq & {∥\mu_{u}-\mu^{*}∥}^{2}+\varrho_{u}^{2}{∥\mu_{u}-\mu_{u-1}∥}^{2}+2\varrho_{u}∥\mu_{u}-\mu^{*}∥∥\mu_{u}-\mu_{u-1}∥ & \\ & & -\lambda (1-\lambda ){∥\beta_{u}-Z\beta_{u}∥}^{2}+2\delta_{u}\langle g\left(\mu^{*}\right)-\mu^{*},\mu_{u+1}-\mu^{*}\rangle , & \end{array}$$

which yields


$$\begin{array}{cccc} \lambda (1-\lambda ){∥\beta_{u}-Z\beta_{u}∥}^{2} & \leq & {∥\mu_{u}-\mu^{*}∥}^{2}-{∥\mu_{u+1}-\mu^{*}∥}^{2} & \\ & & +\varrho_{u}^{2}{∥\mu_{u}-\mu_{u-1}∥}^{2}+2\varrho_{u}∥\mu_{u}-\mu^{*}∥∥\mu_{u}-\mu_{u-1}∥ & \\ & & +2\delta_{u}\langle g\left(\mu^{*}\right)-\mu^{*},\mu_{u+1}-\mu^{*}\rangle . \end{array}$$ (21)

Letting *u* → *∞* and using the conditions lim ⁡ u→∞δu=0 and lim ⁡ u→∞ϱuδu ∥μu−μu−1∥=0, we obtain that


lim ⁡ u→∞ ∥βu−Zβu∥2=0. (22)

Further,


$$\begin{array}{cccc} ∥\gamma_{u}-\beta_{u}∥ & = & ∥(1-\lambda )\beta_{u}+\lambda Z\beta_{u}-\beta_{u}∥ & \\ & = & \lambda ∥\beta_{u}-Z\beta_{u}∥\to 0\text{ as }u\to \infty , \end{array}$$ (23)

$$\begin{array}{rcll} ∥\ell_{u}-\beta_{u}∥ & = & ∥\rho Z\gamma_{u}-\beta_{u}∥ & \\ & \leq & ∥\gamma_{u}-\beta_{u}∥\to 0\text{ as }u\to \infty , & \end{array}$$

and


$$\begin{array}{cccc} ∥\alpha_{u}-\mu_{u}∥ & = & ∥\mu_{u}+\varrho_{u}\left(\mu_{u}-\mu_{u-1}\right)-\mu_{u}∥ & \\ & = & \varrho_{u}∥\mu_{u}-\mu_{u-1}∥\to 0\text{ as }u\to \infty . \end{array}$$ (24)

Consider


$${∥\beta_{u}-\mu^{*}∥}^{2}=∥A_{\varphi_{P}}^{B_{P}}\left(\Phi^{P-1}\alpha_{u}\right)-\mu^{*}∥.$$

Since, $A_{\varphi_{P}}^{B_{P}}$ is firmly nonexpansive, then


$$\begin{array}{llll} {∥\beta_{u}-\mu^{*}∥}^{2} & \leq \langle \Phi^{P-1}\alpha_{u}-\mu^{*},\beta_{u}-\mu^{*}\rangle \; & & \;\\ & =\frac{1}{2}{∥\Phi^{P-1}\alpha_{u}-\mu^{*}∥}^{2}+\frac{1}{2}{∥\beta_{u}-\mu^{*}∥}^{2}-\frac{1}{2}{∥\Phi^{P-1}\alpha_{u}-\beta_{u}∥}^{2},\; & & \; \end{array}$$

which implies that


$$\begin{array}{rcll} & & {∥\beta_{u}-\mu^{*}∥}^{2} & \\ & \leq & {∥\Phi^{P-1}\alpha_{u}-\mu^{*}∥}^{2}-{∥\Phi^{P-1}\alpha_{u}-\beta_{u}∥}^{2} & \\ & \leq & {∥\Phi^{P-2}\alpha_{u}-\mu^{*}∥}^{2}-{∥\Phi^{P-2}\alpha_{u}-\Phi^{P-1}\alpha_{u}∥}^{2}-{∥\Phi^{P-1}\alpha_{u}-\beta_{u}∥}^{2} & \\ & \leq & {∥\Phi^{P-3}\alpha_{u}-\mu^{*}∥}^{2}-{∥\Phi^{P-3}\alpha_{u}-\Phi^{P-2}\alpha_{u}∥}^{2} & \\ & & -{∥\Phi^{P-2}\alpha_{u}-\Phi^{P-1}\alpha_{u}∥}^{2}-{∥\Phi^{P-1}\alpha_{u}-\beta_{u}∥}^{2} & \end{array}$$

Following the same approach, we get


$$\begin{array}{cccc} {∥\beta_{u}-\mu^{*}∥}^{2} & \leq & {∥\alpha_{u}-\mu^{*}∥}^{2}-\overset{P}{\underset{k=1}{\sum }}{∥\Phi^{k-1}\alpha_{u}-\Phi^{k}\alpha_{u}∥}^{2} & \\ & \leq & {∥\mu_{u}-\mu^{*}∥}^{2}+\varrho_{u}^{2}{∥\mu_{u}-\mu_{u-1}∥}^{2}+2\varrho_{u}∥\mu_{u}-\mu^{*}∥∥\mu_{u}-\mu_{u-1}∥ & \\ & & -\overset{P}{\underset{k=1}{\sum }}{∥\Phi^{k-1}\alpha_{u}-\Phi^{k}\alpha_{u}∥}^{2}. \end{array}$$ (25)

It follows from (16), (17), (18) and (25) that


$$\begin{array}{rcll} & & {∥\mu_{u+1}-\mu^{*}∥}^{2} & \\ & \leq & \delta_{u}b_{1}{∥\mu_{u}-\mu^{*}∥}^{2}+(1-\delta_{u}){∥\gamma_{u}-\mu^{*}∥}^{2}+2\delta_{u}\langle g\left(\mu^{*}\right)-\mu^{*},\mu_{u+1}-\mu^{*}\rangle & \\ & \leq & \delta_{u}b_{1}{∥\mu_{u}-\mu^{*}∥}^{2}+(1-\delta_{u}){∥\beta_{u}-\mu^{*}∥}^{2}+2\delta_{u}\langle g\left(\mu^{*}\right)-\mu^{*},\mu_{u+1}-\mu^{*}\rangle & \\ & \leq & \delta_{u}b_{1}{∥\mu_{u}-\mu^{*}∥}^{2} & \\ & & +(1-\delta_{u})[{∥\mu_{u}-\mu^{*}∥}^{2}+\varrho_{u}^{2}{∥\mu_{u}-\mu_{u-1}∥}^{2} & \\ & & +2\varrho_{u}∥\mu_{u}-\mu^{*}∥∥\mu_{u}-\mu_{u-1}∥-\overset{P}{\underset{k=1}{\sum }}{∥\Phi^{k-1}\alpha_{u}-\Phi^{k}\alpha_{u}∥}^{2}] & \\ & & +2\delta_{u}\langle g\left(\mu^{*}\right)-\mu^{*},\mu_{u+1}-\mu^{*}\rangle & \\ & \leq & \left[1-\delta_{u}(1-b_{1})\right]{∥\mu_{u}-\mu^{*}∥}^{2}+\varrho_{u}^{2}{∥\mu_{u}-\mu_{u-1}∥}^{2} & \\ & & +2\varrho_{u}∥\mu_{u}-\mu^{*}∥∥\mu_{u}-\mu_{u-1}∥ & \\ & & -\overset{P}{\underset{k=1}{\sum }}{∥\Phi^{k-1}\alpha_{u}-\Phi^{k}\alpha_{u}∥}^{2}+2\delta_{u}\langle g\left(\mu^{*}\right)-\mu^{*},\mu_{u+1}-\mu^{*}\rangle , & \end{array}$$

which yields,


$$\begin{array}{cccc} \overset{P}{\underset{k=1}{\sum }}{∥\Phi^{k-1}\alpha_{u}-\Phi^{k}\alpha_{u}∥}^{2} & \leq & {∥\mu_{u}-\mu^{*}∥}^{2}-{∥\mu_{u+1}-\mu^{*}∥}^{2} & \\ & & +\varrho_{u}^{2}{∥\mu_{u}-\mu_{u-1}∥}^{2}+2\varrho_{u}∥\mu_{u}-\mu^{*}∥∥\mu_{u}-\mu_{u-1}∥ & \\ & & +2\delta_{u}\langle g\left(\mu^{*}\right)-\mu^{*},\mu_{u+1}-\mu^{*}\rangle . \end{array}$$ (26)

In (26), taking *u* → *∞* and using the conditions lim ⁡ u→∞δu=0 and lim ⁡ u→∞ϱuδu ∥μu−μu−1∥=0, we obtain that


lim ⁡ u→∞∑k=1P ∥Φk−1αu−Φkαu∥2=0.

Hence,


lim ⁡ u→∞ ∥Φk−1αu−Φkαu∥2=0. (27)

Also, we have


$$\begin{array}{ccc} ∥\beta_{u}-\alpha_{u}∥\leq ∥\Phi^{P}\alpha_{u}-\Phi^{P-1}\alpha_{u}∥+∥\Phi^{P-1}\alpha_{u}-\Phi^{P-2}\alpha_{u}∥+\cdots +∥\Phi^{1}\alpha_{u}-\alpha_{u}∥. & & \end{array}$$ (28)

From (27) in (28) after taking the limit as *u* → *∞* ,  we have


lim ⁡ u→∞ ∥βu−αu∥=0. (29)

It follows from (24) and (29) that


$$\begin{array}{ccc} ∥\mu_{u}-\beta_{u}∥\leq ∥\mu_{u}-\alpha_{u}∥+∥\alpha_{u}-\beta_{u}∥\to 0\text{ as }u\to \infty . & & \end{array}$$ (30)

From the definition of $\mu_{u+1},$ and since lim ⁡ u→∞δu=0, we conclude that


$$\begin{array}{cccc} ∥\mu_{u+1}-\ell_{u}∥ & = & ∥\delta_{u}g\left(\mu_{u}\right)+(1-\delta_{u})\ell_{u}-\ell_{u}∥ & \\ & = & ∥\delta_{u}g\left(\mu_{u}\right)-\delta_{u}\ell_{u}∥ & \\ & = & \delta_{u}∥g\left(\mu_{u}\right)-\ell_{u}∥\to 0. \end{array}$$ (31)

Based on (23), (24), (29), (31), we can write


lim ⁡ u→∞ ∥μu+1−μu∥=0.

From the inequalities (22), (24), (27), (30), and Lemma 3.3, we have


limsup ⁡ u→∞⟨g (μ∗)−μ∗,μu−μ∗⟩≤0. (32)

Using (20) and (32) and Lemma 2.9, we obtain that $\mu_{u}\to \mu^{*}.$

Case 2: Assume that for any $u_{0}\in \mathbb{N}$, the sequence $\{t_{u}\}\subseteq \mathbb{R}$ is not a monotonic decreasing. Then, there is a sequence $\left\{u_{b}\right\}$ of  { *u* }  in order that $t_{u_{b}}\leq t_{u_{b}+1}$ for each *b* ∈ *ℕ* .  Inequality (21) implies that


$$\begin{array}{rcll} \lambda (1-\lambda ){∥\beta_{u_{l}}-Z\beta_{u_{l}}∥}^{2} & \leq & {∥\mu_{u_{l}}-\mu^{*}∥}^{2}-{∥\mu_{u_{l}+1}-\mu^{*}∥}^{2} & \\ & & +\varrho_{u_{l}}^{2}{∥\mu_{u}-\mu_{u_{l}-1}∥}^{2}+2\varrho_{u_{l}}∥\mu_{u_{l}}-\mu^{*}∥∥\mu_{u_{l}}-\mu_{u_{l}-1}∥ & \\ & & +2\delta_{u_{l}}\langle g\left(\mu^{*}\right)-\mu^{*},\mu_{u_{l}+1}-\mu^{*}\rangle . & \end{array}$$

Using the conditions lim ⁡ u→∞δu=0 and lim ⁡ u→∞ϱuδu ∥μu−μu−1∥=0, we have


lim ⁡ l→∞ ∥βul−Zβul∥=0.

So,


lim ⁡ l→∞ ∥γul−βul∥= lim ⁡ l→∞λ ∥βul−Zβul∥=0,

and


lim ⁡ l→∞ ∥ℓul−βul∥= lim ⁡ l→∞ ∥ρZγul−βul∥=0.

Utilizing (26), we have


$$\begin{array}{rcll} \overset{P}{\underset{k=1}{\sum }}{∥\Phi^{k-1}\alpha_{u_{l}}-\Phi^{k}\alpha_{u_{l}}∥}^{2} & \leq & {∥\mu_{u_{l}}-\mu^{*}∥}^{2}-{∥\mu_{u_{l}+1}-\mu^{*}∥}^{2} & \\ & & +\varrho_{u_{l}}^{2}{∥\mu_{u_{l}}-\mu_{u_{l}-1}∥}^{2}+2\varrho_{u_{l}}∥\mu_{u_{l}}-\mu^{*}∥∥\mu_{u_{l}}-\mu_{u_{l}-1}∥ & \\ & & +2\delta_{u_{l}}\langle g\left(\mu^{*}\right)-\mu^{*},\mu_{u_{l}+1}-\mu^{*}\rangle . & \end{array}$$

Letting *l* → *∞* in the above inequality, we get


lim ⁡ l→∞∑k=1P ∥Φk−1αul−Φkαul∥2=0⇒ lim ⁡ l→∞ ∥Φk−1αul−Φkαul∥2=0.

Analogously as in Case 1, one can write


lim ⁡ l→∞ ∥βul−αul∥=0,lim ⁡ l→∞ ∥μul+1−γul∥=0,

and


lim ⁡ l→∞ ∥μul+1−μul∥=0.

Using the same method as in Case 1, we conclude that


limsup ⁡ l→∞⟨g (μ∗)−μ∗,μul−μ∗⟩≤0. (33)

Analogue to the inequality (20), we have


$$\begin{array}{ccc} t_{u_{l}+1}\leq (1-q_{u_{l}})t_{u_{l}}+q_{u_{l}}r_{u_{l}}, & & \end{array}$$ (34)

hence


$$q_{u_{l}}t_{u_{l}}\leq q_{u_{l}}r_{u_{l}}.$$

Since $q_{u_{l}}>0,$ we get $t_{u_{l}}\leq r_{u_{l}}.$ Therefore,


$$\begin{array}{cccc} ∥\mu_{u_{l}}-\mu^{*}∥ & \leq & \frac{2}{1-b_{1}}\langle g\left(\mu^{*}\right)-\mu^{*},\mu_{u_{l}+1}-\mu^{*}\rangle +\frac{\varrho_{u_{l}}^{2}}{\delta_{u_{l}}(1-b_{1})}{∥\mu_{u_{l}}-\mu_{u_{l}-1}∥}^{2} & \\ & & +\frac{2\varrho_{u_{l}}}{\delta_{u_{l}}(1-b_{1})}∥\mu_{u_{l}}-\mu^{*}∥∥\mu_{u_{l}}-\mu_{u_{l}-1}∥. \end{array}$$ (35)

It follows from (33) and (35) that lim ⁡ l→∞ ∥μul−μ∗∥=0. By inequalities (33) and (34), we have lim ⁡ l→∞ ∥μul+1−μ∗∥=0. Moreover, since $∥\mu_{l}-\mu^{*}∥\leq ∥\mu_{u_{l}+1}-\mu^{*}∥$ for all *l* ∈ *ℕ*, we get $\mu_{u}\to \mu^{*}.$ This completes the proof.

## 4 Applications

No one can deny the importance of algorithms in scientific applications, especially in the field of optimization. Therefore, in this section we apply the main results to find the solution of the split minimization problem (SMP) and split feasibility problems (SFP) as applications.

### 4.1 Application to the SMP

In this part, we proposed the following SMP:


Find μ∈ℜ such that f (μ)∈ min ⁡ β∈ℜf (β), (36)

where the function *f* : *ℜ* → ( − *∞* , *∞* ]  is a convex, proper and lower semicontinuous (CPLS, for short).

The subdifferential of a function is defined as

**Definition 4.1.** [44] Assume that *f* : *ℜ* → ( − *∞* , *∞* ]  is a CPLS function. The subdifferential *∂f* of *f* is defined by


∂f(β)= {γ∈ℜ:f(β)−f(μ)≥⟨β−μ,γ⟩ for all β,μ∈ℜ}.

Minty [45] proved that the subdifferential *∂f* : *ℜ* → *ℜ* is MMO on *ℜ* ,  while Le [46] showed that the problem (36) is equivalent to the problem


Find μ∈ℜ such that 0∈∂f(β).

Based on the above facts and using Theorem 3.4, we can introduce the following theorem:

**Theorem 4.2.** Let $Q_{j}:ℜ\to ℜ$ be a finite class of $\phi_{j}$-generalized DMMs where $\phi_{j}\in (0,\infty )$ and $I-Q_{j}$ is demiclosed at the origin for all *j* = 1 , 2 , ⋯ , *u* and $f_{k}:ℜ\to (-\infty ,\infty ]$ be a CPLS functions for all *j* = 1 , 2 , ⋯ , *P* .  Let *g* be a $b_{1}$-contraction mapping with $b_{1}\in [0,1)$ and $\Theta_{SMP}=\left\{\cap_{j=1}^{u}fix(Q_{j})\right\}\cap \left\{\cap_{k=1}^{P}\partial f_{k}^{-1}(0)\right\}\neq \emptyset .$ For any $\mu_{1}\in ℜ,$ assume that the sequence $\left\{\mu_{u}\right\}$ is described by


$$\{\begin{array}{c} \alpha_{u}=\mu_{u}+\varrho_{u}\left(\mu_{u}-\mu_{u-1}\right),\text{ where }\varrho_{u}=\{\begin{array}{c} \min\left\{\frac{\tau_{u}}{∥\mu_{u}-\mu_{u-1}∥}\right\},\text{ if }\mu_{u}\neq \mu_{u-1},\\ 0,\text{ otherwise,} \end{array}\\ \beta_{u}=A_{\varphi_{P}}^{\partial f_{P}}A_{\varphi_{P-1}}^{\partial f_{P-1}}\cdots A_{\varphi_{1}}^{\partial f_{1}}\alpha_{u},\\ \gamma_{u}=(1-\lambda )\beta_{u}+\lambda \left(\overset{u}{\underset{j=1}{\sum }}{𝜗}_{j}\left[\left(1-b\right)I+bQ_{j}\right]\beta_{u}\right),\\ \ell_{u}=\rho \left(\overset{u}{\underset{j=1}{\sum }}{𝜗}_{j}\left[\left(1-b\right)I+bQ_{j}\right]\beta_{u}\right)\gamma_{u},\\ \mu_{u+1}=\delta_{u}g\left(\mu_{u}\right)+(1-\delta_{u})\ell_{u}, \end{array}$$

where $\Theta_{SMP}$ refers to the solution of the problem (36), *b* ∈ ( 0 , *λ* )  with *λ* ∈ ( 0 , 1 ) .  Further $\phi_{j}b>0$, ${\left\{\delta_{u}\right\}}_{u=1}^{\infty },$ ${\left\{{𝜗}_{u}\right\}}_{u=1}^{\infty },$ and *ρ* ∈ ( 0 , 1 )  under the following assumptions:

(i) $\sum_{j=1}^{\infty }\delta_{u}=\infty ,$ lim ⁡ u→∞δu=0,
(ii) for a positive term sequence $\left\{\tau_{u}\right\}$, lim ⁡ u→∞τuδu=0.

Then, $\mu_{u}\to \mu^{*}\in P_{\Theta_{SMP}}g(\mu^{*})\in \Theta_{SMP}.$

**Proof.** The result is obtained immediately from Theorem 3.4 by applying the knowledge that *∂f* : *ℜ* → *ℜ* is a MMO on *ℜ* and entering $\partial f_{k}=B_{k}$ for all *k* = 1 , 2 , ⋯ , *P* .  □

### 4.2 Application to the SFP

We discuss the following SFP in this part, which was created by Elfving and Censor [47]:


$$\begin{array}{ccc} \text{Fid }\mu \in M\text{ such that }E\left(\mu \right)\in M, & & \end{array}$$ (37)

where *M* is NCC of *ℜ* and *E* : *ℜ* → *ℜ* is a bounded linear operator. The inductor of the set *M* is described as


$$i_{M}(\varrho )=\{\begin{array}{cc} 0, & \text{if }\varrho \in C,\\ \infty , & \text{otherwise.} \end{array}$$

The formula for a normal cone of M at *ϱ* ∈ *ℜ* is given by


$$\Psi_{M}(\varrho )=\left\{\Upsilon \in ℜ:\langle \Upsilon ,𝜗-\varrho \rangle \leq 0\right\},\text{ for all }𝜗\in ℜ.$$

Obviously, $i_{M}$ is CPLS function and $\partial i_{M}$ is MMO. Moreover,


$$A_{\varphi }^{\partial i_{M}}\left(\varrho \right)={\left(I+\varphi \partial i_{M}\right)}^{-1}\left(\varrho \right),\text{ for all }\varrho \in ℜ,$$

where $A_{\varphi }^{\partial i_{M}}$ is the resolvent operator associated with $\partial i_{M}$ and *φ* > 0 .  Further,


$$A_{\varphi }^{\partial i_{M}}\left(\varrho \right)⟺P_{M}\left(\varrho \right)\text{ for all }\varrho \in ℜ,\varphi >0.$$

According the above illustrations and using Theorem 3.4, we present the following theorem:

**Theorem 4.3.** Let $Q_{j}:ℜ\to ℜ$ be a finite class of $\phi_{j}$-generalized DMMs where $\phi_{j}\in (0,\infty )$ and $I-Q_{j}$ is demiclosed at the origin for all *j* = 1 , 2 , ⋯ , *u*. Assume that $E_{k}:ℜ\to ℜ$ is a bounded linear operator for all *j* = 1 , 2 , ⋯ , *P* .  Let *g* be a $b_{1}$-contraction mapping with $b_{1}\in [0,1)$ and $\Theta_{SFP}=\left\{\cap_{j=1}^{u}fix(Q_{j})\right\}\cap \left\{\cap_{k=1}^{P}P_{M}(0)\right\}\neq \emptyset .$ For any $\mu_{1}\in ℜ,$ assume that the sequence $\left\{\mu_{u}\right\}$ is given by


$$\{\begin{array}{c} \alpha_{u}=\mu_{u}+\varrho_{u}\left(\mu_{u}-\mu_{u-1}\right),\text{ where }\varrho_{u}=\{\begin{array}{c} \min\left\{\frac{\tau_{u}}{∥\mu_{u}-\mu_{u-1}∥}\right\},\text{ if }\mu_{u}\neq \mu_{u-1},\\ 0,\text{ otherwise,} \end{array}\\ \beta_{u}=P_{M_{P}}\left(\varrho \right)P_{M_{P-1}}\left(\varrho \right)\cdots P_{M_{1}}\left(\varrho \right)\alpha_{u},\\ \gamma_{u}=(1-\lambda )\beta_{u}+\lambda \left(\overset{u}{\underset{j=1}{\sum }}{𝜗}_{j}\left[\left(1-b\right)I+bQ_{j}\right]\beta_{u}\right),\\ \ell_{u}=\rho \left(\overset{u}{\underset{j=1}{\sum }}{𝜗}_{j}\left[\left(1-b\right)I+bQ_{j}\right]\beta_{u}\right)\gamma_{u},\\ \mu_{u+1}=\delta_{u}g\left(\mu_{u}\right)+(1-\delta_{u})\ell_{u}, \end{array}$$

where $\Theta_{SMP}$ refers to the solution of the problem (37), *b* ∈ ( 0 , *λ* )  with *λ* ∈ ( 0 , 1 ) .  Further, $\phi_{j}b>0$, ${\left\{\delta_{u}\right\}}_{u=1}^{\infty },$ ${\left\{{𝜗}_{u}\right\}}_{u=1}^{\infty },$ and *ρ* ∈ ( 0 , 1 )  under the following assumptions:

(i) $\sum_{j=1}^{\infty }\delta_{u}=\infty ,$ lim ⁡ u→∞δu=0,
(ii) for a positive term sequence $\left\{\tau_{u}\right\}$, lim ⁡ u→∞τuδu=0.

Then, $\mu_{u}\to \mu^{*}\in P_{\Theta_{SFP}}g(\mu^{*})\in \Theta_{SFP}.$

**Proof.** The result is obtained immediately from Theorem 3.4 by applying the knowledge that $\partial i_{M}$ is MMO on *ℜ* and entering $\partial i_{M_{k}}=B_{k}$ for all *k* = 1 , 2 , ⋯ , *P* .  □

## 5 Conclusion and future work

This manuscript presents a novel algorithm for analyzing strong convergence of sequences under specific conditions, incorporating generalized demimetric operators in real HSs. The algorithm effectively resolves split minimization and feasibility problems. Our research offers a versatile tool for optimization and FP analysis, providing a unified approach to tackle complex problem. With robust convergence guarantees, this method ensures reliable results in practical applications across various fields, including applied mathematics, optimization, and engineering. Future research directions can further enhance our algorithm’s robustness and applicability. Key avenues include investigating adaptive techniques for real-time parameter adjustment, expanding the methodology to non-HSs, applying it to high-dimensional optimization problems, and conducting empirical studies in real-world applications such as data analysis, machine learning and control systems to inform future improvements.

## Abbreviations

MMO: maximal monotone operator.
HS: Hilbert space.
FP: fixed points.
DMM: demimetric mapping.
NCC: non-empty, convex and closed.
MM: Maximal monotone.
SMP: split minimization problem.
CPLS: convex, proper and lower semicontinuous.
