# Expression of Concern: Creating a novel algorithm for studying the strong convergence to a sequence with applications

**DOI:** 10.1371/journal.pone.0343283

**Published:** 2026-02-19

**Authors:** The PLOS One Editors

The *PLOS One* Editors issue this Expression of Concern because this article [1] was identified as one of a series of submissions for which we have concerns about the peer review process. Readers are advised to interpret the article [1] with caution.
